# How to Develop an Electronic Clinical Endometriosis Research File Integrated in Clinical Practice

**DOI:** 10.1155/2015/460925

**Published:** 2015-07-09

**Authors:** A. Vanhie, A. Fassbender, D. O, C. Tomassetti, C. Meuleman, K. Peeraer, S. Debrock, Th. D'Hooghe

**Affiliations:** ^1^Leuven University Endometriosis Center of Expertise, University Hospital Leuven, Herestraat 49, 3000 Leuven, Belgium; ^2^Department of Obstetrics and Gynaecology, Leuven University Fertility Center, University Hospital Leuven, Herestraat 49, 3000 Leuven, Belgium; ^3^Department of Development and Regeneration, Faculty of Medicine, Leuven University, University Hospital Leuven, Herestraat 49, 3000 Leuven, Belgium; ^4^Leuven University Fertility Center, 3000 Leuven, Belgium; ^5^Faculty of Medicine, Leuven University, 3000 Leuven, Belgium; ^6^Faculty of Medicine, Yale University, New Haven, CT, USA; ^7^International Advisory Board, Institute of Primate Research (WHO CC), Nairobi 00502, Kenya

## Abstract

Endometriosis is associated with a range of pelvic-abdominal pain symptoms and infertility. It is a chronic disease that can have a significant impact on various aspects of women's lives, including their social and sexual relationships, work, and study. Despite several international guidelines on the management of endometriosis, there is a wide variety of clinical practice in the management of endometriosis, resulting in many women receiving delayed or suboptimal care. In this paper we discuss the possibilities and benefits of using electronic health records for clinical research in the field of endometriosis. The development of a wide range of clinical software for electronic patient records has made the registration of large datasets feasible and the integration of research files and clinical files possible. Integration of global standards on registration of endometriosis care in electronic health records could improve reporting of research data and facilitate the execution of large, multicentre randomized trials on the management of endometriosis. These highly needed trials could bring us the evidence needed for the optimisation of management of women with endometriosis.

## 1. Introduction

Endometriosis is a chronic, oestrogen-dependent inflammatory condition characterized by the presence of endometrial-like tissue outside the uterus [1]. The WERF EndoCost study has shown that the cost arising from women with endometriosis treated in referral centres is substantial, resulting in an economic burden that is at least comparable to the burden associated with other chronic diseases, like diabetes mellitus [2]. The clinical importance of endometriosis is also reflected by the immense number of publications on this subject. A Medline search for “endometriosis,” on 1 October 2014, gives more than 20 000 results, with more than 800 papers published per year since 2009.

This large body of scientific research in endometriosis has led to important advances in the understanding of the aetiology and pathogenesis of endometriosis, and these novel insights hold promise for the development of new diagnostic and therapeutic approaches for the future [3]. Nevertheless, some key questions in the management of endometriosis remain unanswered. A noninvasive diagnostic test with high sensitivity and specificity is lacking, and there are few or insufficient data from randomized trials to inform the optimal management of endometriosis [4]. The 2013 ESHRE Guideline on management of women with endometriosis emphasizes the need for optimisation of management of women with endometriosis because there is a wide variety of clinical practice in the management of endometriosis, resulting in many women receiving delayed or suboptimal care [2].

Since early development in the 1960s, there has been a steady evolution towards the replacement of the paper based medical records by electronic health records (EHR). During the last 5–10 years, there has been a very rapid expansion to the point where now, in some countries, nearly 90% of all health records are digital [5]. The past decades of progress in health information technology have reshaped the way health care is carried out and how health data are being documented [5]. Research and development projects are ongoing in several countries around the world on the development of infrastructure for national health information, the use of patient generated health data in clinical practice, and the use of EHR data in clinical research [5–7]. At present, healthcare practice stores huge amounts of patient-specific information in EHRs and databases [5]. The use of these electronic health data holds great promise to contribute to improving clinical practice and medical research, to support health care planning and to facilitate quality control of medical care.

In this paper, we discuss the possibilities and benefits of using electronic health records for clinical research in the field of endometriosis. In the first part, we discuss the key elements that should be included in electronic health records from the perspective of clinical research needs in endometriosis. In the second part, we describe how electronic health records could be optimally designed for the integration of clinical care and research. In the last two paragraphs we discuss the costs, possible savings, benefits, and limitations of implementing integrated research files and EHRs on a large scale.

## 2. Necessary Data from a Research Perspective

In the mid-1990s, in response to concerns about quality of reporting data from RCTs, an international group developed the Consolidated Standards of Reporting Trials (CONSORT) statement [8]. The CONSORT statement, published in 1996 and revised in 2001, is a set of guidelines designed to improve the reporting of RCTs [8, 9]. In a systematic review, the beneficial effect on reporting RCTs by adoption of the CONSORT criteria has been demonstrated [10]. Since 1996, the CONSORT statement has been extended to RCTs of nonpharmacologic treatments [11]. Furthermore, additional guidelines have been published for other types of studies, for example, the STROBE-statement (STrengthening the Reporting of OBservational studies in Epidemiology) for observational studies and the PRISMA-statement (Preferred Reporting Items for Systematic Reviews and Meta-Analyses) for systematic reviews and meta-analyses [12, 13].

Endometriosis has a significant effect on various aspects of women's lives, including their social and sexual relationships, work, and study [2]. Women with endometriosis can have a range of pelvic-abdominal pain symptoms, including dysmenorrhoea, dyspareunia, heavy menstrual bleeding, nonmenstrual pelvic pain, pain at ovulation, dyschezia, and dysuria, as well as chronic fatigue [1]. Endometriosis is also associated with infertility, with a strong association between severity of disease and impact on fertility [1].

For research databases and randomised trials, well-characterised and relevant outcome measures are very important. Recommendations for pain scoring in clinical trials on endometriosis have been published but they are lacking other important aspects of the disease [14]. For example, the lack of international agreement on terms and definitions to assess clinical outcome in the surgical treatment of endometriosis has led to the use of a multitude of different outcome measures and the lack uniformity in the reporting of surgical data. In a systematic review of 2011 on the surgical management of deep endometriosis with colorectal extension, comparison between surgical techniques was impossible due to incomplete and inadequate reporting in the trials under investigation [15]. Out of 49 studies included in that review, only one study reported data on all the outcome variables under review (complications, pain, quality of life, fertility, and recurrence) [15]. In another systematic review of 2010 on bowel resection for deep endometriosis, meta-analysis was again impossible due to lack of uniformity in reporting of data [16].

Recently the World Endometriosis Research Foundation (WERF) has launched the WERF Endometriosis Phenome and Biobanking Harmonisation Project (EPHect) [17–21]. The purpose of EPHect is to facilitate and enable large-scale, cross-centre, longitudinal, epidemiologically robust, biomarker and treatment target discovery research in endometriosis through the development of a consensus on detailed clinical phenotyping (phenome) data to be collected from women with endometriosis and on standard operating procedures (SOPs) for banking of biological samples from women with endometriosis and controls, with respect to collection, transport, processing, and long-term storage [17–21]. The global consensus of detailed phenotypic characterization and standard operating procedures is anticipated to provide a platform to interpret biochemical, genetic/epigenetic, genomic, and quality of life data relevant to endometriosis symptoms and targeted therapies [17–21]. Projects such as the WERF EPHect are very important to standardize reporting in endometriosis research and similar, international, initiatives with detailed guidelines for standardised reporting of other aspects of endometriosis research, such as the medical and surgical treatment of endometriosis, are needed to improve and harmonize reporting.

In conclusion, it can be stated that an ideal clinical endometriosis research file incorporates outcome measures of all relevant aspects of endometriosis. Detailed guidelines for data to be collected in clinical endometriosis research, such as the EPHect, should be developed for all aspects of endometriosis research and form the basis of any endometriosis research file. Furthermore attention should be paid to report data according to international standards such as CONSORT.

## 3. Electronic Health Record (EHR): Integration of Research and Clinical Practice

Several studies have demonstrated that implementing an EHR can yield real benefits in terms of increased delivery of care based on guidelines, enhanced monitoring and surveillance activities, reduction of medication errors, and decreased rates of utilization for potentially redundant or inappropriate care [22]. The success of EHRs depends on the quality and completeness of the information available to health care professionals in making decisions about patient care and in the communication between health care professionals during patient care. It is important therefore to assess the data quality if information is entered in electronic systems by different health care professionals [6].

The main challenge in clinical practice is how relevant and essential clinical research items for endometriosis can be integrated in an electronic health record which remains user friendly for daily clinical practice. To use EHR systems efficiently for clinical research, a number of features are required that, unfortunately, have not yet been implemented. Functions are required to ensure the correctness, completeness, and accuracy of the data within the EHR systems; there also has to be a structured or coded use of nomenclature to allow data extraction [5]. So far, EHRs have largely consisted of unstructured, narrative text and to a small extent of structured coded data. In the future it will be necessary to implement more systematic terminologies and codes so that the data contained in EHRs can be put to better use [6]. Two widely used clinical healthcare terminology databases are the ICD (International Classification of Diseases) and SNOMED-CT (systematized nomenclature of medicine clinical terms) developed and supported by the WHO (World Health Organization) and IHTSDO (International Health Terminology Standards Development Organisation), respectively [23, 24]. Using standardized nomenclature in EHRs is important from a research perspective: the development of strategies for automatic or convenient use of this nomenclature is essential for the integration of routine clinical practice and scientific research. Furthermore, the consistent use of standardized nomenclature not only facilitates data extraction from EHRs, but also creates the possibility to exchange, integrate, and compare data from different EHRs. This “semantic interoperability” is on top of the Health Informatics agenda. It targets the preservation of meaning between heterogeneous patient-related and aggregated population data across different vocabularies and coding systems. In order to meet this demand, the WHO and IHTSDO have decided to create a Common Ontology [25].

## 4. Benefits and Limitations of EHR Use in Routine Clinical Practice and Clinical Research

The introduction from an electronic health record has several benefits in comparison to a paper based patient record. The EHR enables centralized collection of data instead of the fragmented paper records due to patient care provided at multiple locations [26]. Furthermore EHRs can decrease medical errors, facilitate detection of adverse health events, and increase the safety of the process of giving medications [26]. Finally the EHR has helped quality control and health care planning through the possibility of evaluation of care based on date extracted from patient records [26].

The next challenge is to adapt these clinical EHRs for use in clinical research, reducing the need to develop separate clinical research databases that require duplication of patient data. High quality research requires very rigorous registration of clinical data from the first patient contact till long term follow-up after treatment. A possible method is to integrate selectively clinical research in an existing EHR. In the context of endometriosis, this could be done by integrating general clinical data, as outlined in general guidelines for clinical research (i.e., CONSORT and STROBE), and endometriosis-specific data, as presented in endometriosis-specific guidelines. In Tables 1 and 2 a summary of recommendations for the reporting of medical and surgical trials in endometriosis is presented [14, 27]. In Table 3 an overview of the WERF EPHect project is provided. Since it is impossible to record too many research data in clinical practice, choices have to be made by each research group about essential and optional items. Ideally, a link should be constructed between the EHR and direct web based reporting of patient reported outcomes. This will become increasingly important in long term follow-up of patients with endometriosis, in order to address the problem of low response rates, leading to substantial amounts of missing data and selection bias.

## 5. Costs and Possible Savings of an Electronic Research File

The potential health and financial benefits of widespread implementation of health information technology (HIT) in the US has been estimated to be more than $81 billion annually, by improving health care efficiency and safety [28]. However, this estimation has been challenged, since these potential savings have not been realized in the USA [29] or in the UK [30, 31].

This inability to realise financial benefits through the implementation of HIT can be explained largely by inadequate management and development strategies by the IT industry [29]. Indeed, few health IT vendors make products that are easy to use, resulting in complaints from many doctors and nurses that health IT systems slow them down [29].

In contrast, EHR systems can be very successful if they are developed in collaboration with medical staff and regularly adapted based on feedback from clinical practice [28, 29]. This strategy makes the EHR-system more user friendly and improves quality of care and patient outcomes at a significantly lower cost than most electronic health care systems [29].

At present, no data are available about cost and possible cost savings from an electronic research file integrated within the EHR used in daily practice. From our own experience we believe that several important factors need to be taken into account in cost calculations for an electronic clinical research file. First of all, there is the time spent by researchers extracting their data from patients files. As described above, data in EHRs are very rarely coded and as such automated data extraction is not possible. In the context of case control trials, retrospective evaluation of EHRs is needed in order to retrieve essential and relevant clinical research information from cases and controls, a very time consuming and frustrating process. In the context of prospective cohort studies, this situation leads to double registration of clinical data by the medical staff on the one hand and the researchers on the other hand. Secondly, the extra time required to enter data for research purposes in EHRs can lead to improvement in their accuracy and completeness and serve the quality of clinical care as well.

In our hospital we have chosen to incorporate as many research data as possible in our electronic health record used in daily practice. Based on suggestions and feedback from both researchers and medical staff we have tried to minimize the burden of extra work/time and maximize the gains for both clinical care and research aims. We estimate that more detailed clinical records and facilitation of data extraction from this clinical database for research will be cost effective, in the sense that that the extra time spent by the medical staff will be compensated by the time gained in data extraction for research or quality control purposes. Our effort is also expected to facilitate retrospective studies and data registration for our Endometriosis Biobank [32].

## 6. Conclusion 

The technological advancements of the last decades in EHRs have supported improvement of clinical care and research. These new technologies have facilitated the analysis of health care processes and influenced medial decision making, health care planning, and medical research. The development of a wide range of clinical software for electronic patient records has enabled the registration of large datasets and the integration of medical records with clinical research files. A fast and user friendly system is needed to enter clinical research data and patient reported outcomes in EHRs, linking clinical practice to clinical research in an online modality. General (CONSORT, STROBE, …) and endometriosis specific (WERF EPHECT, …) international standards are needed to decide which data should be collected for which type of research. Ideally all data should automatically be coded in internationally used clinical healthcare terminology and be subject to quality control tools assessing completeness, accuracy, and other dimensions.

## Figures and Tables

**Table 1:** Recommendations for clinical trials in endometriosis.

*Entry criteria *	
Surgical diagnosis of endometriosis in the last 5 years	
Pain symptoms	
Data capture at baseline:	
(i) ASRM staging	
(ii) baseline pain scores over at least 2 menstrual cycles	
(iii) EHP-30	
(iv) previous treatments and responses	

*Primary outcome measures *	
Daily ratings of pelvic pain	
Daily ratings of dysmenorrhea	
Ratings on an 11-point NRS	

*Secondary outcome measures *	
B & B with separate scores for each domain, administered weekly for 6 weeks, then monthly until 6 months, and then at 9, 12, 18, and 24 months	
EHP-30 with separate and total scores, administered at the same tie points as the B & B	
Use of rescue analgesia/therapies including an NRS before us and a record of the indication	
Study specific adverse event questionnaires with direct questions and free text, administered at the same time points as the B & B	
Detailed information as per the CONSORT guidelines, including	
(i) the recruitment process	
(ii) the number of participants who were excluded and why	
(iii) the number of candidates who chose not to enter the trial and why	
(iv) the use of prohibited concomitant mediations and other protocol deviations	
(v) the number and reasons for withdrawal from each treatment group	
(vi) the types rates and reasons for nonadherence with treatment in each group	

*Tertiary outcome measures *	
Daily NRS of three symptoms the patient feels are important for her, for example, dyspareunia, dyschezia, fatigue, and so forth	

Adapted from Vincent et al., 2010 [14].

B & B = Biberoglu and Behrman.

NRS = numerical rating scale.

**Table 2:** Recommendations for designing and reporting studies in the surgical treatment of DIE.

*Title and abstract *	
Study type	Clearly define the study type (e.g., prospective, retrospective)

*Introduction *	
Background	Scientific background and explanation of rationale

*Methods *	
Participants	Previous therapeutic surgery: type (diagnostic, therapeutic), number, laparoscopy or laparotomy, endometriosis-related or not
	Indication for surgery: pain, child wish completed, child wish uncompleted, child wish absent
	Sample size and power calculation

Interventions	Endometriosis staging according to ASRM classification; operation time; length of hospital stay; multidisciplinary team including details on which surgeon did which surgery; clear description of the surgical technique according to the following definitions: shaving: superficial peeling of bowel serosal and subserosal endometriosis (with diathermy or laser), superficial excision: selective excision of the bowel endometriosis lesion without opening of the bowel wall, full thickness disc excision: selective excision of the bowel endometriosis lesion with opening followed by closure of the bowel wall, and bowel resection anastomosis: resection of a bowel segment affected by endometriosis followed by anastomosis report type and number of concomitant procedures in detail

Follow-up period	Define the period of follow-up (in months)
	Details on the follow-up procedure (e.g., telephone interview, questionnaire, and clinical evaluation)
	Patients lost during follow-up period

Pain measurement	Define the method used for pain measurement: presurgery and postsurgery, number of patients using hormonal treatment at the time of pain assessment, 11-point numerical scale for the assessment of menstrual pain (dysmenorrhea), nonmenstrual pain, dyspareunia; use of other methods (interviews, questionnaires): provide full details.
	Patient-based or doctor-based

QOL measurement	Define the method used for QOL measurement (e.g., EHP-30, SF-36, and EQ-5D)

Fertility rate	Number of patients with history of infertility
	Number of patients wishing to conceive passively (wish for reservation/restoration of fertility during surgery, without well-defined child wish at the time of surgery); number of patients wishing to conceive actively with a well-defined child wish in the near future; number of patients wishing to conceive actively with a well-defined child wish in the distant future

Recurrence rate	Define recurrence: (1) symptom recurrence based on patient history, but no proof of recurrence by imaging and surgery; (2) endometriosis recurrence based on imaging: in patients with or without symptoms (pain and infertility). Recurrence is then likely based on noninvasive imaging (e.g., ultrasound and MRI); (3) surgical reintervention without recurrence of endometriosis: in patients with recurrent symptoms, surgery without visual diagnosis of endometriosis, and with either normal pelvis or other abnormalities (e.g., adhesions); (4) recurrence of visual endometriosis without histological proof: during laparoscopy endometriosis is visually observed but either not biopsied or biopsied without histologically proven endometriosis; (5) recurrence of histologically proven endometriosis: during laparoscopy endometriosis is visually observed and confirmed histologically. Suspicious recurrent endometriosis is present if the criteria for categories 1 and 2 were met. Proven recurrent endometriosis is present if the criteria for categories 4 and 5 were met. Additional surgery without evidence for endometriosis is present if the criteria for category 3 are met.

Statistical methods	Statistical methods used; life table analysis methods; handling of patients lost for follow-up

*Results *	
Histological confirmation	Report degree of endometriosis invasion in bowel
	Report the median length of the resected colorectal segments (in cm)
	Report the median largest diameter of the lesions (in cm)
	Report the number of positive margins over the number of resected bowel specimens; report the number of patients with at least one positive margin of the bowel resection specimen
Complications	Report all major complications and their clinical management [surgery (specify type of surgery), medical, and expectant] including rectovaginal fistulae, anastomotic leaks, postoperative stomas, abscesses, and postoperative bleedings in absolute numbers

Fertility rate	Report cumulative pregnancy rate (life table analysis)
	Number of women who conceived
	Median time to conceive after surgery
	Mode of conception: spontaneous or medically assisted conception (ovulation induction; intrauterine insemination with or without ovarian stimulation; assisted reproduction: IVF and ICSI; fresh cycle or cryocycle; egg reception or embryo reception)
	Live birth rate; ectopic pregnancy rate, miscarriage rate, and clinical pregnancy rate

Recurrence rate	Report cumulative recurrence rate (life table analysis)

*Discussion *	
Interpretation	Interpretation of the results, taking into account study hypotheses, sources of potential bias or imprecision, and the dangers associated with the multiplicity of analyses and outcomes.

Generalizability	External validity of the trial findings

Overall evidence	General interpretation of the results in the context of current evidence

Adapted from Meuleman et al., 2012 [27].

**Table 3:** Overview of WERF EPHect.

Project I	**Surgical phenotype data collection in endometriosis research**
	Provides a standard recommended and a minimum required surgical form (SSF and MSF) to collect data on the surgical phenotype of endometriosis.

Project II	**Clinical and covariate phenotype data collection in endometriosis research**
	Provides a standard recommended and minimum required self-administered endometriosis patient questionnaire (EPQ) to capture detailed clinical and covariate data.

Project III	**Fluid biospecimen collection, processing, and storage in endometriosis research**
	Provides recommended and minimum required standard operating procedures for biofluid collection, processing, and storage in endometriosis research.

Project IV	**Tissue collection, processing, and storage in endometriosis research**
	Provides standard recommended and minimum required standard operating procedures for tissue collection, processing, and storage in endometriosis research.

Summary of Becker et al., 2014 [17], Vitonis et al., 2014 [18], Rahmioglu et al., 2014 [19], and Fassbender et al., 2014 [20].
